# Biopsychosocial, work-related, and environmental factors affecting work participation in people with Osteoarthritis: a systematic review

**DOI:** 10.1186/s12891-023-06612-6

**Published:** 2023-06-13

**Authors:** Angela Ching, Yeliz Prior, Jennifer Parker, Alison Hammond

**Affiliations:** Centre for Human Movement and Rehabilitation, University of Salford, Salford, Greater Manchester UK

**Keywords:** Osteoarthritis, Employment, Absenteeism, Presenteeism, Work loss

## Abstract

**Purpose:**

Osteoarthritis (OA) causes pain and disability, with onset often during working age. Joint pain is associated with functional difficulties and may lead to work instability. The aims of this systematic review are to identify: the impact of OA on work participation; and biopsychosocial and work-related factors associated with absenteeism, presenteeism, work transitions, work impairment, work accommodations, and premature work loss.

**Methods:**

Four databases were searched, including Medline. The Joanna Briggs Institute Critical Appraisal tools were used for quality assessment, with narrative synthesis to pool findings due to heterogeneity of study designs and work outcomes.

**Results:**

Nineteen studies met quality criteria (eight cohort; 11 cross-sectional): nine included OA of any joint(s), five knee-only, four knee and/or hip, and one knee, hip, and hand OA. All were conducted in high income countries. Absenteeism due to OA was low. Presenteeism rates were four times greater than absenteeism. Performing physically intensive work was associated with absenteeism, presenteeism, and premature work loss due to OA. Moderate-to-severe joint pain and pain interference were associated with presenteeism, work transition, and premature work loss. A smaller number of studies found that comorbidities were associated with absenteeism and work transitions. Two studies reported low co-worker support was associated with work transitions and premature work loss.

**Conclusions:**

Physically intensive work, moderate-to-severe joint pain, co-morbidities, and low co-worker support potentially affects work participation in OA. Further research, using longitudinal study designs and examining the links between OA and biopsychosocial factors e.g., workplace accommodations, is needed to identify targets for interventions.

**Systematic review registration:**

PROSPERO 2019 CRD42019133343.

**Supplementary Information:**

The online version contains supplementary material available at 10.1186/s12891-023-06612-6.

## Background

Osteoarthritis (OA) is the most common form of arthritis [1]. Incidence and prevalence are higher in women, with the most affected joints (in order) being the knee, hip, wrist/hand then ankle/foot joints [2, 3]. Age is a strong risk factor [4], with prevalence increasing between 40 to 44 years of age in women and 45 to 49 years in men [2, 5, 6]. Those overweight or obese have nearly three times the risk of knee OA compared to those of normal weight, and this is a modifiable risk factor [4].

Work participation (i.e., being employed/in paid work) is increasingly seen as a primary outcome of rehabilitation [7]. OA often starts when people are still employed [8–10], with a higher prevalence in those whose work involves repeated squatting, kneeling, and/or heavy lifting [9–11]. OA leads to joint pain and reduced function, affecting occupational performance [8] and leading to work instability (i.e., a mismatch between functional capacity and work demands which can threaten employment if not resolved [12]. This is associated with absenteeism (taking sick days off work), presenteeism (reduced work productivity at work), work transitions (work interruptions due to a health condition), work impairment (factors that reduce work ability/capacity), and premature work loss due to ill-health [13–17]. These impact on individuals’ home life, daily activities, quality of life and have financial consequences for the individual and society [18]. However, the use of workplace accommodations (defined as organisation-level practices that may be used by employees to accommodate their work and health needs, such as flexible hours; special equipment or adaptations (e.g., ergonomic chairs or equipment); or modified work schedules (e.g., more breaks) have been shown to improve employment outcomes in employed people with OA or inflammatory arthritis compared to those who do not use these accommodations [19].

Biopsychosocial and work-related factors can help explain the impact of OA on individuals’ work. The World Health Organization’s International Classification of Functioning, Disability and Health (ICF) provides a framework for measuring health and disability in individuals and the population [20]. Heerkens et al*.,* have extended the ICF to classify how an individual’s work functioning, work activities, and participation can be affected by their: 1) health or disease; 2) external factors, such as work-related factors (relationships, tasks, employment conditions), work load (mental and physical), non-work related load (e.g., family/caring responsibilities), other external factors (e.g., home/social support); and 3) personal factors, such as a person’s functional capacity (physical and mental), work-related personal factors (e.g., motivation to work harder) and general personal factors (e.g., age, sex, education, self-efficacy, coping) [21].

Two systematic reviews evaluated studies published to 2013 of the effects of OA on work [8, 22]. There was a mild negative effect of OA on work participation (i.e., having paid work, work productivity, absenteeism, work disability, or early retirement); even though people experienced work problems, only a small proportion left work as a result [8]. However, the authors noted that evidence was sparse to support conclusions [8]. Chronic knee pain or knee OA were strongly associated with absenteeism but there was limited evidence for effects on presenteeism, as only one cohort study examined this [22]. At the time, there was little evidence available about which individual or work-related factors are associated with absenteeism and none available evaluating which factors affect presenteeism in people with chronic knee pain or knee OA [22]. Work accommodations can help people stay in work; employed people with rheumatoid arthritis (RA) with workplace accommodations are 2.5 times more likely to remain in work [23]. However, there were few studies investigating workplace accommodations outcomes in employed people with OA [8]. In the last 10 years, further research has been published meaning that the impact of contextual factors on work in OA can now be investigated.

The impact of OA on work participation is growing due to an ageing population and the obesity epidemic [24]. Additionally, the increasing State Pension age means people living with OA will need to stay in the workforce for longer. The aims of this systematic review were to identify and summarise the impact of OA on work participation and the biopsychosocial and work-related factors associated with absenteeism, presenteeism, work transitions, work impairment, work accommodations, and premature work loss.

## Methods

### Protocol and registration

The review protocol was registered with PROSPERO (registration number: PROSPERO 2019 CRD42019133343) and is available to view at: https://www.crd.york.ac.uk/prospero/display_record.php?ID=CRD42019133343

### Literature search

Studies were identified by searching four electronic databases: Allied and Complementary Medicine (Ovid; 1985–May 2022); The Cumulative Index to Nursing and Allied Health Literature (EBSCOhost; 1976–May 2022); MEDLINE (Ovid; 1946–May 2022); and APA PsycInfo (Ovid; 1806–May 2022). The search strategy was developed using medical subjects heading (MeSH) terms and text words related to OA, absenteeism, presenteeism, work impairment, productivity, and biopsychosocial factors that may impact work participation. (See Additional File 1 for the Medline Ovid search strategy).

### Eligibility criteria

Publication date or publication status restrictions were not imposed.

#### Types of studies

Observational studies, e.g., cohort, cross-sectional, and case–control, assessing work participation in people with OA. Interventional and qualitative studies were excluded.

#### Types of participants

Adults (aged 18 years or over); OA in any joint(s) (diagnosed radiographically or clinically, physician-diagnosed, or participant self-reported); OA as the primary condition perceived as the main impact on work; self-employed or in paid employment at least one day/ week; and may or may not have had joint replacement surgery due to OA.

#### Context

Any setting.

#### Outcome measures

Prevalence of at least one of: absenteeism; presenteeism; work impairment; work transition; premature work loss (i.e., due to ill-health); workplace accommodations.

### Study selection

**Fig. 1:**
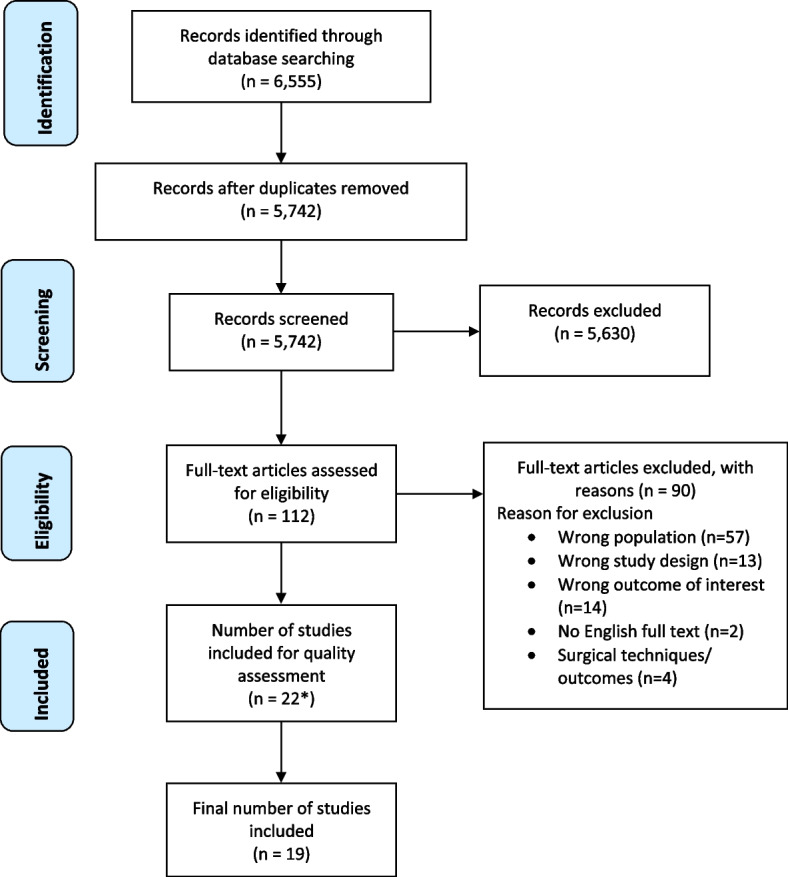
PRISMA flow diagram of records identified, screened, assessed for eligibility, and included. *See Additional File 3 for reasons of exclusion of 3 papers at quality assessment stage

Study titles and abstracts were retrieved, then screened independently by two reviewers (AC, YP) to identify those meeting eligibility criteria. Eligible full-text articles were then independently screened (AC, YP) for inclusion (Fig. 1). Disagreements were resolved by discussion (AC, YP) and if no agreement reached, discussed with a third reviewer (AH).

### Data extraction

A data extraction form was developed and pilot-tested on five randomly selected studies and refined accordingly. One reviewer (AC) extracted data from included studies. The lead author of one study was contacted for numerical data for OA-only participants [25]. Data extracted included: study characteristics, participant characteristics, outcome measures, and study results. For the full data items extracted, please see the protocol registered with PROSPERO.

### Methodological quality assessment

Four reviewers (AC, YP or AH, JP) critically appraised study methodological quality using the Joanna Briggs Institute for cohort or cross-sectional studies, as appropriate (see Additional File 2) [26]. Studies scoring ≤ 50% (low quality) were excluded and those scoring 51–79% (moderate quality) and 80–100% (good quality) were included [26]. Disagreements were resolved through discussion with all four reviewers to reach a consensus.

## Results

### Study selection

The PRISMA flow diagram is shown in Fig. 1. Twenty-two articles were initially identified as eligible and quality assessed, with three then excluded [27–29] (see Additional File 3 for exclusion reasons).

### Synthesis of results

A narrative synthesis of findings was conducted due to the heterogeneity in study designs and work outcomes. Study characteristics, e.g., study design; participant demographics; OA joint(s); study size and setting; and outcome measures reported are included in Table 1. Additional Files 4 and 5 are tables summarising findings for the following outcomes (where available): absenteeism; presenteeism; work impairment; work transitions; and premature work loss. Additional File 6 summarises workplace accommodation outcomes.

**Table 1:** Characteristics of included studies

Author, year, country	Study aims	Study design (follow-up) and data	Study population	Diagnosis of osteoarthritis (OA)	Joint(s) with OA	N; Participants’ details (age, sex)	Relevant outcome measures
***Cohort Studies***							
Agaliotis et al*.*, 2013, Australia [24]	To determine: (1) reduced work productivity (absenteeism/ presenteeism) and (2) associated risk factors, in symptomatic knee OA	1 year Q + D	Older workers (aged ≥ 45 years); completing 2-year “Long-term Evaluation of Glucosamine Sulfate” study	Diagnosis based on medial tibiofemoral joint space narrowing (≥ 2 mm joint space) on x-ray	Knee	*n* = 360 Mean age (SD) 57.5 (7.2) y 54.0% F	Work productivity: 1) Absenteeism (past 2 months): How many days off due to knee problems? 2) Presenteeism (daily for 1 week): self-reported ‘estimated capacity from 0% (unable to do usual work/ activities) to 100% (fully functioning in usual role).’ Questions derived from WPAI:OA
Hubertsson et al*.*, 2017, Sweden [30]	To determine association between occupation and risk for absenteeism and disability pension due to knee or hip OA	5-years R	Residents Skåne region, Sweden; Skåne Health Care Register data linked with SSIA data	ICD-10 code for knee OA (M17); hip OA (M16)	Knee and hip	*n* = 165,179 Age: 40-70y.; (average age not reported) 58.3% F	Absenteeism and disability pension data from SSIA register
Kontio et al*.*, 2018, Finland [31]	To examine association of education and physical workload factors with occupational differences in disability retirement due to knee OA	8-years R	Population-based; register data from 70% random sample of Finnish population; aged 18–70 years living in Finland on 31/12/2004 (~ 2.5 million)	Finnish version ICD-10 (1996) code for knee OA (M17)	Knee	*n* = 1,135,654 Mean age (SD): F = 45.3 (8.4) y; M = 44.6 (8.3) y 49.4% F	Full-time disability retirement (either temporary or permanent) due to knee OA from 01/01/2005 to 31/12/2013
Kontio et al*.*, 2020, Finland [32]	To examine to what extent disabling OA, led to prolonged absenteeism, interferes with work participation and shortens working life-years	8-years R	Population-based study; register data from 70% random sample of Finnish population; aged 18–70 years living in Finland on 31/12/2004 (~ 2.5 million)	Finnish version ICD-10 (1996) codes: M15 (polyarthritis), M16 (hip OA), M17 (knee OA), M18 [carpometacarpal (CMC) joint OA, and M19 (other OA) For the analysis, CMC joint OA and polyarthritis were combined	Hip, knee, hand and other	*n* = 4,704 Mean age (SD): Knee OA = 51.1 (6.2); Hip OA = 52.0 (6.0) Polyarthritis or CMC joint OA = 53.5 (4.6); Other OA = 51.1 (6.7) 56.2% F	Sustained return to work (i.e., returning to regular duties for ≥ 28 consecutive days immediately following sickness absence). Time spent in different work participation statuses Potential working life–years lost (i.e., actual retirement age (whatever cause) versus working life expectancy forecast for Finland). Early exit from paid employment (i.e., permanent disability retirement or old age retirement prior to 63 years old)
Summanen et al*.*, 2021, Finland [33]	To determine the burden of hip and knee OA in Finnish occupational healthcare	8-years R	Electronic medical records (EMRs) of Terveystalo (largest private and occupational healthcare provider in Finland)	Diagnosis of: hip or knee OA (ICD-10 codes M16* or M17*, respectively)	Hip and knee	*N* = all OA cohort: 51,068; OCH subcohort from all OA cohort = 35,109; controls = 35,101 Mean (SD) age at index: ALL OA = 56.6 (10.1); OCH subcohort = 53.3 (7.7); Controls = 52.4 (7.6) F: all OA cohort = 56.3%; OCH subcohort hip and knee = 54.2%; Controls = 54.2%	Absenteeism days
Wilkie et al*.*, 2014, United Kingdom [34]	To estimate proportion of working age adults with OA predicting work limitations prior to future pension age of 69	6-years Q	North Staffordshire OA project (NorStOP): population-based prospective cohort study	At least one consultation during study period for OA based on NHS Read codes (N05 category) for primary care consultations	Any joint(s) – not specified	*n* = 297. consulters for OA Mean age (SD) 54.0 (2.34) y 54.9% F	Expected work limitations: at 6-y. follow-up, “Do you think joint pain will limit your ability to work before you reach 69 years old” (will limit or stop me/ don’t know/ won’t limit)
Wilkie et al*.*, 2014, United Kingdom [17]	To describe the extent of premature work loss and associated factors in OA consulters	6-years Q	NorStOP – a population-based prospective cohort study	At least one consultation during the study period primarily for OA based on NHS Read codes (N05 category) for primary care consultations	Any joint(s) – not specified	*n* = 612. consulters for OA Mean age (SD) 54.6 (2.8) y 48.2% F	Premature work loss (i.e., moving from employment to retirement prior to state retirement age or transition from employment to being off work due to ill-health or unemployment)
Wilkie et al*.*, 2015, United Kingdom [35]	To examine how pain leads to onset of work productivity loss in OA; and identify new intervention opportunities	3-years Q	NorStOP—population-based prospective cohort study	At least one consultation during study period for OA based on NHS Read Codes (N05 category) for primary care consultations	Any joint(s) – not specified	*n* = 318 Mean age (SD): 56.2 (2.2) y 52.8% F	Work productivity loss (SF-36 item: “During the past 4 weeks, have you accomplished less than you would like in your work or other regular daily activities as a result of your physical health? yes/no) Pain intensity (SF-36 Bodily Pain in last 4 weeks: high (i.e., moderate, severe, very severe) or low (i.e., none, very mild, mild)
***Cross-Sectional Studies***							
Agaliotis et al*.*, 2017, Australia [36]	To explore personal and workplace environmental factors as predictors of reduced worker productivity among older workers with chronic knee pain	Q + D	Older workers (aged ≥ 45 years) completing 2-year “Long-term Evaluation of Glucosamine Sulfate” study	Knee pain, or taking NSAIDs/ analgesia for knee pain on most days in the past month; Knee pain of 4–10 on 10 cm Visual Analogue Scale; Medial tibio-femoral compartment joint space narrowing in symptomatic knee	Knee	*n* = 129 Mean age (SD) 60.0 (6.8) y 52.0% F	Single-item questions from WPAI:OA for absenteeism (past 2 months) and presenteeism (7-day diary); Multi-item at-work limitations/productivity = Work Transition scale (past 6 months); Workplace Activity Limitations Scale
Bieleman et al*.*, 2010, The Netherlands [13]	To examine work participation of people with early knee or hip OA and compare to data from American Osteoarthritis Initiative (OAI) cohort. The influence of health status and personal factors on work participation analysed	Q	The Cohort Hip and Cohort Knee (CHECK) study; data from the OAI	**CHECK:** Self-reported pain and/ or stiffness hip and/or knee; first GP consult for these symptoms ≤ 6 m; OA explained their symptoms. **OAI:** Baseline data participants’ clinical and joint status; risk factors for progression and development of knee OA (questionnaires and examination)	Knee and hip	**Participants in paid work:** **CHECK**: *n* = 970 Mean age (SD) 56.0 (6.0) y 79% F **OAI**: *n* = 1,578. Mean age (SD) 56.0 (6.0) y 64% F	Work participation (including: present or last job, work hours, working history, present working status, absenteeism) investigated using “Economic Aspects in Rheumatoid Arthritis” questionnaire. Labour force participation (i.e., paid job ≥ 8 h/ week) For participants in paid employment: present health condition; whether adapted, or like to adapt, their work (tasks/hours/ workplace). Participants not in paid work: why not employed?
Conaghan et al*.*, 2021, Europe [37]	To assess whether impact of OA pain on health-related quality of life, work, and healthcare resource use differed by pain severity and prescription medication status	NS	National Health and Wellness Survey (NHWS) 2016–2017 (pooled self-reported data) from the 5 European countries	Self-reported physician-diagnosed OA; experienced pain in past 12 months (worst pain with or without prescription medication use [none/mild/moderate/severe])	Any joint(s) – top 3 affected joints: knees, fingers, hips	*n* = 2,417 Age groups (years), n (%): 18-39y = 94 (3.9%); 40-49y = 236 (9.8%); 50-59y = 550 (22.8%); 60-69y = 940 (38.9%); 70 + y = 597 (24.7%) 64.5% F	WPAI: GH (work productivity loss; non-work activity impairment). HRQoL and health status: SF-12v2; EQ-5D; SF-6D; EQ-VAS. All-cause healthcare resources utilization past 6 months: self-reported no. healthcare provider visits (primary care, emergency/ urgent care, and hospitalisations)
daCosta DiBonaventura et al*.*, 2011, USA [38]	To evaluate impact of OA pain on: direct medical costs; indirect costs associated with lost productivity for any reason; healthcare provider visits; and general health status in employed population	NS	NHWS 2009	Self-reported physician-diagnosed OA	Ankles, elbows, feet, fingers, hands, hips, knees, neck, shoulders, spine, wrists, other	*n* = 2,173. diagnosed with OA Age range: 20–39 y.: *n* = 267; 40–64 years *n* = 1,453; ≥ 65 y. *n* = 453 56.1% F	Work productivity (WPAI. Health utility scores (using SF-6D). For each respondent, percent overall work impairment (WPAI) multiplied by annual income. Direct and indirect costs summed to estimate total costs
daCosta DiBonaventura et al*.*, 2012, USA [39]	To evaluate impact of patient-rated OA severity on: productivity; health-related quality of life (HRQoL); healthcare resource use and costs in employed individuals compared to employed individuals without OA	NS	NHWS 2009	Self-reported physician-diagnosed OA;	Ankles, elbows, feet, fingers, hands, hips, knees, neck, shoulders, spine, wrists, other	Total *n* = 4,876 **Mild OA** *n* = 2,192, Age range: 20-39y. *n* = 338; 40–64 y. *n* = 1,339; ≥ 65 y. *n* = 515 48.9% F; **Moderate OA** *n* = 2,240 Age range: 20–39 y. *n* = 298; 40-64y. *n* = 1,447; ≥ 65 y. *n* = 495 55.0% F; **Severe OA** *n* = 444; Age range: 20–39 y. *n* = 49; 40–64 y. *n* = 312; ≥ 65 y. *n* = 83. 57.2% F	Work productivity (WPAI). HRQoL (physical and mental component scores SF-12v2). Health utility scores (SF-6D)
Gignac et al*.*, 2018, Canada [25]	To compare health and work contexts of M:F workers ≥ 50 years to understand similarities/ differences in workplace accommodation availability, need, use, and unmet needs	Q	Born 1946 to 1964; employed ≥ 15 h/week. Data from larger project on health, accommodations, and retirement expectations in arthritis, diabetes, no chronic disabling health conditions	Self-reported physician diagnosis of arthritis; duration ≥ 1 year (to ensure has worked with arthritis). **OA only data used in this systematic review**	Any joint(s) – not specified	*n* = 273 Mean age: F = 59.5 y M = 59.1 y 64.8% F	Fourteen workplace accommodations: availability (Y/N), need for (Y/N), and use of (Y/N) in previous 12 m.; accommodation needs reported as (i) congruence between accommodations needed and used (i.e., accommodation needs met), (ii) needing more accommodations than used (i.e., accommodation needs unmet), or (iii) not needing some accommodations, but using anyway (i.e., accommodation needs exceeded)
Hermans et al*.*, 2012, The Netherlands [40]	1. To identify and quantify knee-related productivity and medical costs in conservatively treated knee OA patients in paid employment 2. To evaluate associations between knee-related productivity loss and individual, disease and work characteristics	Q	Participants in a randomised controlled trial investigating cost-effectiveness of intra-articular hyaluronic acid plus usual care **Only data acquired from baseline measurement (pre-intervention) used**	Consecutive patients with knee OA at 2 hospitals out-patient Orthopaedic clinics; Kellgren/Lawrence grade 1–3 and pain score ≥ 2 (0–10 numerical rating scale); age 18–65 y.; treated conservatively for knee OA for ≥ 6 m. prior to inclusion	Knee	*n* = 117 Mean age (SD) 53.2 (7.4) y 43.0% F	Productivity and medical costs: Productivity and Disease Questionnaire measuring: productivity costs due to knee symptoms; knee-related absence from work past 3 m.; lost productivity due to knee symptoms while at work (using Quality and Quantity method)
Hubertsson et al., 2013, Sweden [14]	To investigate extent of absenteeism and disability pension in knee OA; and compare to general population	Retrospective Secondary data analysis; R	Skåne Health Care Register data linked with SSIA data	Skåne Health Care Register data: diagnosis of knee OA (ICD-10 code M17)	Knee	*n* = 15,345 Mean age (SD): F = 55.0 (8.2) y.; M = 53.0 (9.2) y 49.6% F	Absenteeism and disability pension data retrieved from SSIA register
Jackson et al*.,* 2020, USA and Europe [41]	To evaluate burden of pain associated with knee and/or hip OA	Patient records; Q	Large, multinational, observational study of clinical practice (Adelphi Disease Specific Programme (DSP)™	Patients diagnosed with knee and/or hip OA by consulting physicians	Knee and/or hip	*N* = 2170 Mean age (SD): 66.4 (11.2) y 57.9% F	Work productivity and daily activity impairment (WPAI:OA); HRQoL
Laires et al*.*, 2018, Portugal [42]	To describe impact of OA (pain and physical disability) on early exit from work	Q	National, cross-sectional, population-based study of rheumatic diseases in Portugal – the EpiReumaPt study	Clinical confirmation OA by a rheumatologist; validated by three experienced rheumatologists using American College of Rheumatology criteria	Knee, hip, hand	*n* = 382 Mean age 57.5y 72.3% F	Self-reported employment status: employed (i.e., part/ full-time); early exit from paid employment (including: students; homemakers; no regular salary; early retired; or with disability pension) Quality of life (SF-36v2 total and 8 sub-scale scores); EQ-5D-3L; longstanding musculoskeletal pain (≥ 3 m.); pain interference with function (i.e., pain affecting work and domestic activities from item in SF-36v2; functional capacity (Health Assessment Questionnaire)
Nakata et al*.*, 2018, Japan [43]	To examine work impairment and OA; HRQoL and health status	NS	2014 Japan National Health and Wellness Survey (NHWS, Kantar Health, New York, NY, USA),	Self-reported received OA diagnosis from a healthcare provider	Ankles, elbows, feet, fingers, hands, hips, knees, neck, shoulders, spine, wrists, other	*n* = 233 Mean age (SD): 54.2 (12.2) y 43.8% F	Work productivity (WPAI). Presenteeism and absenteeism categorised as any vs. none. HRQoL (SF-36v2; SF-6D). Depression (Patient Health Questionnaire-9)

Key: Diagnosis: *OA* Osteoarthritis, *ICD10* International Classification of Diseases, Tenth Revision; Study data: *R* Register; *Q* Questionnaire, *NS* National Survey, *D* Diary, *DB* Database(s); Data source, *SSIA* Swedish Social Insurance Agency, *NorStOP* North Staffordshire OA project, *CHECK* the Cohort Hip and Cohort Knee, *OAI* Osteoarthritis Initiative, *NHWS* National Health and Wellness Survey, *Gender F/M* Female / Male, *Y* Years; Measures: *WPAI-OA* Work Productivity and Activity Impairment Questionnaire: osteoarthritis of the knee or hip v2.0, *SF-6D* = Medical Outcomes Survey Short-Form Six-Item, *Medical Outcomes Survey SF-12v2* Short-Form 12-item (v2), *SF-36 v2* Medical Outcomes Study Short Form 36 Health Survey (v2), *EQ-5D-5 (or 3)L* EuroQol 5 dimension 5 level questionnaire. Other: *vs* versus, *GP* general practitioner, *NHS* National Health Service in the United Kingdom, *HRQoL* Health-related quality of life, *NSAIDs* Non-steroidal anti-inflammatory drugs, *SD* Standard deviation

### Study characteristics

Of the 19 studies, eight were cohort [17, 24, 30–35] and 11 cross-sectional studies [13, 14, 25, 36–43]. Nine reported about OA of any joint(s) [17, 25, 32, 34, 35, 37–39, 43]; five knee OA only [14, 24, 31, 36, 40]; four knee and/or hip OA [13, 30, 33, 41] and one study assessed people with at least one of knee, hip and/or hand OA [42] (Table 1).

Nine studies were based on four datasets: the North Staffordshire Osteoarthritis Project (NorStOP), a population-based prospective cohort study [17, 34, 35]; United States 2009 National Health and Wellness Survey (NHWS [38, 39]; the Long-term Evaluation of Glucosamine Sulfate (LEGS) study [24, 36]; and the Skåne Health Care Register data linked to Swedish Social Insurance Agency data [14, 30]. Amongst the cohort studies, follow-up ranged from one to eight years.

### Methodological quality assessment

Results of the quality assessment are shown in Table 2. All eight cohort studies were good quality. Five cross-sectional studies were moderate and six were good quality.

**Table 2:** Methodological quality assessments of included studies using the Joanna Briggs Institute tools^a^

Author, year, country	1	2	3	4	5	6	7	8	9	10	11	Quality%
**Cohort Studies**												
Agaliotis et al*.*, 2013, Australia [24]	N/A	N/A	Y	Y	Y	N/A	Y	Y	Y	Y	Y	100
Hubertsson et al*.*, 2017, Sweden [30]	Y	Y	Y	Y	Y	N/A	Y	Y	Y	N/A	Y	100
Kontio et al*.*, 2018, Finland [31]	Y	Y	Y	Y	Y	Y	Y	Y	Y	N/A	Y	100
Kontio et al*.*, 2020, Finland [32]	N/A	N/A	Y	Y	Y	Y	Y	Y	Y	N/A	Y	100
Summanen et al*.*, 2021, Finland [33]	Y	Y	Y	Y	Y	Y	Y	Y	Y	N/A	Y	100
Wilkie et al*.*, 2014, United Kingdom [34]	Y	Y	Y	Y	Y	Y	Y	Y	Y	Y	Y	100
Wilkie et al*.*, 2014, United Kingdom [17]	Y	Y	Y	Y	Y	Y	U	Y	Y	U	Y	82
Wilkie et al*.*, 2015, United Kingdom [35]	Y	Y	Y	Y	Y	Y	U	Y	Y	Y	Y	91
**Cross-Sectional Studies**												
Agaliotis et al*.*, 2017, Australia [36]	Y	Y	U	Y	Y	Y	Y	Y	-	-	-	88
Bieleman et al*.*, 2010, The Netherlands [13]	Y	Y	Y	Y	Y	Y	Y	Y	-	-	-	100
Conaghan et al*.*, 2021, Europe [37]	Y	Y	Y	Y	Y	Y	Y	Y	-	-	-	100
daCosta DiBonaventura et al*.*, 2011, USA [38]	Y	Y	N	N	Y	Y	Y	Y	-	-	-	75
daCosta DiBonaventura et al*.*, 2012, USA [39]	Y	Y	U	U	Y	Y	Y	Y	-	-	-	75
Gignac et al*.*, 2018, Canada [25]	Y	Y	Y	U	Y	Y	U	Y	-	-	-	75
Hermans et al*.*, 2012, The Netherlands [40]	Y	Y	Y	Y	Y	Y	Y	Y	-	-	-	100
Hubertsson et al*.*, 2013, Sweden [14]	Y	Y	U	Y	U	U	Y	Y	-	-	-	63
Jackson et al*.*, 2020, USA and Europe [41]	Y	Y	Y	Y	N	N	Y	Y	-	-	-	75
Laires et al*.*, 2018, Portugal [42]	Y	Y	Y	Y	Y	Y	U	Y	-	-	-	88
Nakata et al*.*, 2018, Japan [43]	Y	Y	Y	U	Y	Y	Y	Y	-	-	-	88

Key: ^a^Joanna Briggs Institute (https://jbi.global/critical-appraisal-tools) for cohort or cross-sectional studies; *Y* Yes, *N* No, *U* Unclear, *N/A* Not applicable. Quality scoring: Yes = 1; No = 0; Unclear = 0; N/A = not counted. Quality %: low quality =  ≤ 50%; moderate quality = 51–79%; good quality = 80–100%. See Online Resource 3 for explanation of critical appraisal items (numbers 1–11)

### Work participation outcomes: absenteeism, presenteeism, work impairment and work transitions

Outcomes are summarised in Additional File 4.

#### Absenteeism

Five studies used percentages to report absenteeism due to OA; the rates were between 1.4–14.0% [13, 14, 24, 32, 36]. However, studies measured absenteeism using different timescales, ranging from currently on sick leave to sick leave in the last 12 months, making comparisons difficult.

A large cohort study found hours of work lost due to absenteeism in workers with OA pain was 2.7 (standard deviation (SD) 7.1) hours in the past week, compared to workers without OA pain, losing 1.4 (SD 5.6) hours (*p* < 0.0001) [38]. OA patients had over twice as many days of absenteeism (22.8 vs 8.1 days per patient year (PPY)) and periods of absenteeism (2.2 vs 1.0 PPY) compared to age- and sex-matched controls without OA [33].

Biopsychosocial factors associated with absenteeism were: younger age (any absenteeism = 46.1 (SD 15.3) years versus (vs) none = 55.4 (SD 11.3) years; *p* < 0.001); and a higher comorbidity burden compared to OA patients with no absenteeism (Charlson Comorbidity Index scores: any comorbidity = 3.1 (SD 8.0) vs. none = 0.7 (SD 3.3); p = 0.006) reported in a small cross-sectional study (*n* = 233) [43]. A large Finnish cohort study (*n* = 51,068) analysed data from electronic medical records found that of the 22.8 days of absenteeism PPY for OA patients (vs 8.1 days for controls), 6.3 days of sick leave were recorded as due to OA and 8.4 days due to comorbid conditions [33]. Absenteeism was higher in hip/knee OA patients with type 2 diabetes compared to control patients without diabetes (31.2 vs 7.9 days PPY) [33]. Hip/knee OA patients with chronic obstructive pulmonary disease (COPD) had more days of absenteeism PPY compared to controls without COPD (39.0 vs 8.1 days PPY) [33].

A cross-sectional study (*n* = 2,170) found people with moderate/severe OA pain (score 4–10; Western Ontario and McMaster Universities Osteoarthritis Index (WOMAC) Pain scale) reported greater percentages of work time missed due to health problems than those with no/mild pain (score 0–3) (20.5% vs 5.5% work time missed, respectively) [41]. Additionally, absenteeism was greater in those with, than without, presenteeism (2.9% (SD) 10.8% vs. 0.0% (SD 0.4) %, *p* = 0.03, respectively) [41].

A large Swedish cohort study (*n* = 165,179) found the risk of absenteeism (adjusted for age and education) due to knee OA was three times higher for women working in healthcare (odds ratio (OR): 3.3, 95% confidence interval (CI): 2.6–4.1), childcare (OR: 3.0, 95% CI: 2.3–3.9) and cleaning sectors (OR: 3.1, 95% CI: 2.2–4.2) compared to those in business/administration [30]. For men, it was one to three times higher in farming (OR: 1.7, 95% CI: 1.1–2.5), transport (OR: 1.8, 95% CI: 1.3–2.5), metal work (OR: 2.6, 95% CI: 1.7–3.9), and construction (OR: 3.0, 95% CI: 2.3–3.9), compared to business/administration [30]. Similarly, a small cross-sectional study reported that physically intensive work was significantly associated with absenteeism (OR: 4.2, 95% CI: 1.5–11.9), *p* < 0.05, adjusted for body mass index (BMI) and quality of life [40].

Hip/knee OA patients from a large Finnish cohort with a BMI > 30 kg/m^2^ had more days of absenteeism PPY compared to those with normal BMI (≤ 25 kg/m^2^) (28.2 vs 16.3 days PPY). In controls without OA, there were lower levels of absenteeism but the same trends (9.4 vs 7.3 days PPY for BMI > 30 kg/m^2^ and BMI ≤ 25 kg/m^2^, respectively) [33].

#### Presenteeism

Presenteeism was evaluated by ten studies, using a variety of measures to ascertain productivity loss. The Work Productivity and Activity Impairment (WPAI) questionnaire [44] was used in seven studies [24, 36–39, 41, 43], whilst the Productivity and Disease Questionnaire [40, 45] was used in one cross-sectional study, and cohort study reported on a single item from the Medical Outcomes Study Short Form-36 (SF-36) “During the past 4 weeks, have you accomplished less than you would like in your work or other regular daily activities as a result of your physical health?” [35, 46]. Another cross-sectional study estimated productivity by valuing healthy time lost due to OA using market wage rates in Portugal [42, 47]. This meant that the comparison of presenteeism reported across these studies was challenging.

A cross-sectional study (*n* = 2,173) reported that presenteeism rates and loss of hours due to presenteeism were almost four times greater than for absenteeism [38]. Presenteeism was higher in those with OA pain compared to those without OA or arthritis pain in the past month (31% vs. 16% productive time at work lost (*p* < 0.0001); 9.7 (SD 9.7) hours vs 5.2 (SD 8.6) hours lost (*p* < 0.0001) [38]. Another cross-sectional study (*n* = 2,417) found that OA patients with mild pain treated with prescription medication had a significantly higher level of presenteeism (47.2%) than those with moderate/severe pain untreated with prescription medication (43.9%) (*p* < 0.001) [37]. OA patients in the mild pain treated with prescription medication group had a higher mean number of joints affected by arthritis and more comorbidities compared to those in the moderate/severe pain untreated with prescription medication group.

Furthermore, one cohort study and three cross-sectional studies found that joint pain was significantly associated with reduced work productivity, particularly in those with moderate/severe knee pain in the past week [24, 36, 40, 41]. Similarly, another cohort study reported that high pain intensity at baseline was significantly associated with work productivity loss three years later in OA primary care consulters. This association remained unchanged after adjusting for age, sex, educational attainment, occupational class, and comorbidity [35]. Physical limitation mediated the association between pain intensity and work productivity loss [35].

Findings from cross-sectional studies included in this review reported other biopsychosocial and work-related factors associated with presenteeism included: problems with one or more joints other than the knee [36]; higher use of prescription medication [37, 43]; greater depression severity [43]; lower mental and physical health status scores compared to those without presenteeism [43]; an SF-12 Physical Component Summary score of < 50 at baseline [24]; younger age [43]; performing physically intensive work [40]; semi-manual or manual occupations [24]; and job insecurity [36].

#### Work impairment

Three cross-sectional studies measured work impairment using the WPAI [37, 38, 41]. Workers with OA pain had greater work impairment than those without OA pain (34.4% vs 17.8%, *p* < 0.0001) [38]. People with moderate-to-severe pain, with or without opioid use, had significantly greater overall work impairment (52.3% or 44.6%, *p* < 0.05, respectively) than those with no or mild pain without opioid use (23.8%) [41]. Work impairment was greater in those with, than without, presenteeism (39.5% (SD 25.1) % vs. 0.0% (SD 0.4) %, *p* < 0.001) regardless of pain level or opioid use [41]. Similarly, those with moderate/severe pain treated with prescription medications had two to six times higher impairment compared with those with mild pain untreated with prescription medications (*p* < 0.001) [37]. Those with higher pain intensity or moderate/severe pain on prescription medication (including opioids) had the greatest level of comorbidity, i.e., higher rates of depression or anxiety, osteoporosis, sleep disorders, and chronic low back pain compared to those with less pain or not taking prescription medication [37, 41].

#### Work transitions

A cross-sectional study reported the most common work transitions were: work interrupted for at least 20 min (15%); unable to take on extra projects/responsibilities (11%); and lost time at work (e.g., leaving work early, arriving late or taking an extended lunch break) (9%) [36]. A six-year follow-up Finnish study found on average six transitions (95% CI 5.8–6.0) were made between different work participation statuses per person [32]. The work participation statuses investigated were: being at work; on partial work disability; on sickness absence because of OA; on time-restricted full work disability; unemployed; economically inactive (not at work and not receiving ill health–related or unemployment benefit, or pension); on permanent disability retirement; and reached official retirement age (63 years in Finland) [32]. The most common pathway for those with two transitions was from sickness absence to work, followed by being on full disability retirement or reaching official retirement age [32]. A small cross-sectional study found that biopsychosocial and work-related factors associated with work transitions included: moderate-to-severe knee pain in the past week, a comorbidity score of four or more, or low co-worker support [36].

#### Expected work limitations

A UK-based cohort study measured expected work limitations prior to future pension age (69 years) in OA primary care consulters, using a single question “Do you think joint pain will limit your ability to work before you reach 69 years old?” [34]. Better physical function was highly protective against expected work limitations [34]. Work dissatisfaction and low co-worker support were associated with expected work limitations, although by relatively few respondents (25.8% and 6.7%, respectively) [34].

### Outcomes: leaving work before statutory retirement age

These outcomes are summarised in Additional File 5.

#### Premature work loss / early exit from work

Premature work loss is defined differently in studies. A UK cohort study defined this as either being unemployed, stopped working due to ill-health or retiring prior to State Pension age [17]. Being male, pain interference and low co-worker support were independently associated with premature work loss in OA primary care consulters, after adjusting for potential confounders (e.g., age, sex, and socio-economic factors) [17]. A Finnish cohort study defined premature work loss as early exit from paid employment by transiting to permanent disability retirement or retiring prior to 63 years old [32]. Potential working life-years lost was calculated by using actual premature work loss age and Finnish working life expectancy forecast tables (Years 2006–2014) [32]. People with OA lost 2.1 (95% CI: 2.0–2.2) potential working life–years [32]. A Portuguese cross-sectional study measured early exit from work (i.e., having no paid work, receiving disability pensions or officially early retired) [42] and found knee OA was strongly associated with early exit from work, but not hand or hip OA [42]. Furthermore, those with knee OA with the highest levels of disability and worse pain interference were at a greater risk of early exit from work compared to those without knee OA [42].

#### Disability pension / disability retirement

The number of people with knee OA on disability pensions in Sweden increased with age [14]. Women with knee OA had more days disability pension/year than men (94 vs. 47 days) [14]. The risk of having a disability pension due to knee OA (after adjusting for age and education) was increased for: women in the healthcare, childcare, or cleaning sectors; and for men, the construction, metal work, or transport sectors [30]. A large Swedish cohort study reported that the risk of disability pension due to hip OA was increased in all job sectors for women, as compared to business and administration. For men, risk increased only in the farming sector [30].

A large Finnish registry cohort (*n* = 1,135,654) reported that physical load factors (e.g., heavy physical work, heavy lifting, kneeling or squatting work, sitting, standing or moving) were statistically significantly associated with disability retirement due to knee OA in men and women after adjusting for age [31]. All physical load factors, except sitting, increased risk of disability retirement. However, these risk estimates decreased after further adjusting for education [31]. Observed occupational differences in disability retirement were explained by educational level and mediated by physical workload factors [31]. The risk of disability retirement was highest for those in the following occupations: plumbers, electricians and construction workers [31]. Women in physically demanding occupations (i.e., cleaners, kitchen workers, building caretakers, and assistant nurses) had the highest risk of disability retirement compared to those in professional occupations [31].

### Outcomes: work accommodations

A cross-sectional study reported working fewer hours was the most desired and frequently used workplace accommodation in people with knee and/or hip OA [13]. Others, such as taking frequent short breaks and better dividing of effort during a workday (pacing), were also reported [13]. These outcomes are summarised in Additional File 6.

At 12-month follow-up, 28% (*n* = 99) had made at least one change in their work, the most common being changing occupation (*n* = 43). Some reported increasing (*n* = 21) or decreasing (*n* = 20) workhours.

A cross-sectional study of availability of, need for, and use of 14 workplace accommodations, benefits, and practices in the past 12 months investigated if needs for each were unmet, met, or exceeded [25]. Most of the 14 accommodations were needed by < 25%. Women were more likely to need five or more accommodations compared to men, and more likely to receive help with job tasks compared to men [25].

Use of two to four accommodations (compared to zero or one accommodation) was predicted by greater work activity limitations and health variability [25]. Use of five or more accommodations was predicted by work activity limitations, physical work demands and health variability [25]. Participants with OA whose accommodation needs were exceeded were more likely to report greater job control compared to those with unmet needs [25]. Additionally, those with unmet needs were more likely to work in sales/retail, have less job control and increased work stress, compared to those having accommodation needs met [25].

## Discussion

The findings from our systematic review extend that available from previous reviews, published 10 years ago. These identified mild negative effects of OA on work participation [8], but that there was little research available about individual or work-related factors associated with absenteeism, and none about factors associated with presenteeism in people with OA [22]. Since 2014, more studies investigating factors associated with work participation in OA have been published. Despite the heterogeneity of study methodologies and work outcomes limiting our ability to synthesize the body of literature into specific findings, the studies included in this systematic review highlight that physically intensive jobs were associated with absenteeism, presenteeism, and premature work loss due to ill-health (three cohort studies and one cross-sectional study) [24, 30, 31, 40]. Moderate-to-severe joint pain and pain interference were associated with presenteeism, work transitions, and premature work loss (four cohort and four cross-sectional studies) [17, 24, 34, 35, 38, 39, 41, 42]. Physical limitations and worse physical function scores were associated with presenteeism and expected workplace limitations (two cohort studies) [17, 35]. Some evidence suggests that having comorbidities was associated with absenteeism and work transitions (one cohort and two cross-sectional studies) [33, 36, 43]. Low co-worker support was associated with work transitions and premature work loss (one cohort and one cross-sectional studies) [32, 36].

It has been well established that heavy physical workload is a common occupational risk factor for OA. Heavy physical workload factors, such as recurrent squatting, bending, kneeling, climbing stairs, and loading of the knee, contribute to the development of knee OA [48–50]. Physically intensive work, manual or semi-manual labour sectors, or jobs with heavy physical workload were associated with absenteeism, presenteeism, and premature work loss due to ill-health [24, 30, 31, 40]. The risk of manual workers having disability retirement was strongly attributed to physical heavy workload [32]. This is supported by previous epidemiological evidence that increased risk of disability retirement, earlier retirement, and mortality among workers is associated with physically demanding work [51, 52]. There may be other individual and lifestyle factors affecting premature work loss not reported in the studies in this review. For example, those in non-physically intensive occupations may find it easier to stay in work despite OA, while those in physically intensive roles may have limited work ability due to the nature of their work tasks and environment [31].

People with OA experiencing moderate-to-severe joint pain or high pain intensity have reduced work productivity and greater overall work impairment compared to those with no or mild pain or no OA [24, 34, 35, 38, 39, 41]. Additionally, pain interference with normal work or housework was also associated with premature work loss [17, 42]. Previous research has shown that greater initial pain intensity, pain for longer duration, multisite pain and initial functional limitations are predictors of poor functional outcomes in people with OA [3]. The findings in our review show that physical limitations and worse physical function scores were associated with presenteeism and expected workplace limitations. Physical limitation is a mediator in the association between pain intensity and onset of work productivity loss [35]. Those reporting more difficulty performing work-related tasks (e.g., sitting for long periods, standing, and scheduling demands) experienced greater productivity loss [53], which can lead to increased dependency, emotional distress and reduced self-worth [11]. Improving physical function in patients with higher pain levels could improve work productivity outcomes [35].

Some evidence from two small cross-sectional studies and a large cohort study suggests that comorbidity burden was also associated with absenteeism, work impairment, and work transitions [33, 36, 43]. This supports previous research showing associations between musculoskeletal pain, depression, and high blood pressure with reduced worker productivity [54–56]. Additionally, patients experiencing higher pain intensity and currently using prescription medication have the highest comorbidity burden. This is supported by evidence from previous research demonstrating the gastrointestinal and/or cardiovascular adverse effects of opioids [57] and non-steroidal anti-inflammatory drugs use [37, 58]. This highlights the importance of health care professionals considering possible comorbidities, prescription medication use, and how these may impact on people’s work ability and health.

Two studies reported that low co-worker support was associated with work transitions and premature work loss due to OA and knee problems [35, 36]. Previous research identified a lack of perceived co-worker support being associated with greater job strain and work loss in people with arthritis [59]. The fear of being perceived as receiving special treatment was also an important barrier to requesting workplace accommodations or using available support measures, potentially leading to greater job strain and work loss [60]. Thus, it is important that employers and co-workers are aware of work difficulties experienced by people with long-term health conditions, to enable supportive workplaces meeting the requirements of disability equality legislation to help them stay in work.

Only three studies examined workplace accommodations in people with OA. Working fewer hours was the most needed and used accommodation in those with knee and/or hip OA [8]. However, people with arthritis who worked fewer hours reported greater job strain, possibly due to their arthritis limiting their ability to work longer hours or meeting their work demands [61]. Greater accommodation use was predicted by work activity limitations, physical work demands and health variability [25]. The most common accommodations were flexitime (e.g., flexible start and finish work times), extended health benefits, personal days with pay (e.g., paid leave to attend health appointments and care responsibilities) and working from home [25]. Previous research also reported that lack of workplace accommodations, such as flexible working hours and adapting the work environment, are associated with absenteeism and reduced work productivity [62]. This highlights the importance of considering individuals’ symptoms and working environment to help them meet their work demands. Research about workplace accommodations for working people with OA is sparse and is needed to identify how these can help with job retention.

There are limitations to this review. Fourteen studies used the Kellgren-Lawrence classification of OA or secondary care health professionals to confirm the presence of OA in participants, but five studies only used self-reported physician diagnosis of OA, which may reduce reliability of the findings as not all such participants may have OA. However, self-report is a commonly accepted method of defining OA in epidemiological surveys, as OA can be diagnosed clinically without investigation if a person is 45 years old or over, has activity-related joint pain, and either no or less than 30 min of morning joint-related stiffness [63]. A second limitation is that more than half of the included studies were cross-sectional, meaning the link between exposure and outcome cannot be established. More longitudinal studies are required to investigate the link between OA and work participation. Most studies used self-reported data collection, which is prone to recall, attrition, and selection biases. Five studies from Scandinavia used data from national registries, with large cohort sizes, making their findings more generalisable to Scandinavia [14, 30–33]. All the included studies were from high income countries, which probably have better income support systems, paid sick leave policies and wellbeing policies compared to lower income countries, and these may influence reporting of absenteeism or premature work loss. Those studies measuring presenteeism used different outcome measures making it challenging to accurately compare productivity across studies.

## Implications

Heavy physical workload, physically intensive work, moderate-to-severe joint pain, comorbidities, and low co-worker support are associated with poor work participation outcomes. Improving work ability in people with OA requires a multifactorial approach addressing physical, psychological, socio-environmental, and work-related factors to manage the condition, as well as managing associated co-morbidities. These factors affect economic losses or gains in employees and employers, as most with OA could continue to work, despite persistent symptoms, given the right support [18]. In the UK, the Equality Act [64] requires employers to make reasonable adjustments to accommodate employees with long-term disabilities. More studies are required to assess workplace accommodation needs and workplace adjustments made to understand what can be done to adjust work processes for employees living with OA. There was limited evidence in our review that age was associated with absenteeism. Problems with more than one joint, job insecurity, prescription medication use, and greater depression symptom severity were associated with presenteeism, but this warrants further research due to limited evidence. Additionally, using a standard work outcomes core set is needed to facilitate comparisons between work studies. More studies are also required to investigate and explore other personal and environmental factors related to work which were not reported in our review, in order to understand how these factors affect the decision about work participation in employees living with OA and to identify targets for future interventions.

## Conclusions

This review demonstrated that, although limited evidence, there are moderate-to-good quality studies investigating the impact of OA on work participation, especially in terms of how biopsychosocial and work-related factors influence this. It identified factors associated with work participation (such as physically demanding jobs, experiencing moderate-to-severe joint pain, living with co-morbidities, and low co-worker support), which are worth exploring further to help develop personal and workplace strategies to support work participation in employed people with OA.

## Supplementary Information

**Additional file 1.** Example of the search strategy for MEDLINE Ovid database.

**Additional file 2.** Joanna Briggs Critical Appraisal Tool items: Cohort and Cross-sectional studies.

**Additional file 3.** Excluded studies after the quality assessment process.

**Additional file 4.** Outcomes: Work participation.

**Additional file 5.** Outcomes: leaving work before statutory retirement age.

**Additional file 6.** Outcomes: workplace accommodations and adaptations.

## Data Availability

The data that support the findings of this review will be available from the corresponding author upon reasonable request.

## Abbreviations

BMI: Body mass index
CI: Confidence interval
COPD: Chronic obstructive pulmonary disease
ICF: International Classification of Functioning, Disability and Health framework
LEGS: Long-term Evaluation of Glucosamine Sulfate study
MeSH: Medical subjects heading
NHWS: National Health and Wellness Survey
NorStOP: North Staffordshire Osteoarthritis Project
OA: Osteoarthritis
OR: Odds ratio
PPY: Per patient year
RA: Rheumatoid arthritis
SD: Standard deviation
SF-12: Medical Outcomes Study Short Form-12
SF-36: Medical Outcomes Study Short Form-36
vs: Versus
WOMAC: Western Ontario and McMaster Universities Osteoarthritis Index
WPAI: Work Productivity and Activity Impairment
