# Supplementary material for: Biopsychosocial, work-related, and environmental factors affecting work participation in people with Osteoarthritis: a systematic review
Source: BMC Musculoskelet Disord. 2023 Jun 13;24:485. doi: 10.1186/s12891-023-06612-6 (PMC10262400; doi:10.1186/s12891-023-06612-6)
Supplement: Supplementary file 1 — Additional file 1. Example of the search strategy for MEDLINE Ovid database. [file 12891_2023_6612_MOESM1_ESM.docx]

Article title: Biopsychosocial, work-related, and environmental factors affecting work participation in people with Osteoarthritis: A systematic review.

Journal name: BMC Musculoskeletal Disorders

Authors: Angela Ching^1^, Yeliz Prior^1^, Jennifer Parker^1^, Alison Hammond^1^

Affiliation: ^1^Centre for Health Sciences Research, University of Salford, Salford, Greater Manchester, United Kingdom.

Corresponding Author: Professor Yeliz Prior (email: [y.prior@salford.ac.uk](mailto:y.prior@salford.ac.uk))

**Additional File 1: Example of the search strategy for MEDLINE Ovid database.**

| **The effect of osteoarthritis on work participation: a systematic review** | | |
| --- | --- | --- |
| **Database: MEDLINE (Ovid)**  **Ovid MEDLINE(R) and Epub Ahead of Print, In-Process & Other Non-Indexed Citations, Daily and Versions(R) 1946 to 22.05.2022** | | |
| **Population (P1)** | 1. MeSH HEADING: exp Osteoarthritis/ 2. MeSH HEADING: Joint Diseases/ 3. (joint adj disorder*).mp. [mp=title, abstract, original title, name of substance word, subject heading word, floating sub-heading word, keyword heading word, protocol supplementary concept word, rare disease supplementary concept word, unique identifier, synonyms] 4. MeSH HEADING: exp joints/ or elbow joint/ or exp foot joints/ or exp hand joints/ or exp hip joint/ or exp knee joint/ or shoulder joint/ 5. coxarthrosis.mp. [mp=title, abstract, original title, name of substance word, subject heading word, floating sub-heading word, keyword heading word, protocol supplementary concept word, rare disease supplementary concept word, unique identifier, synonyms] 6. gonarthrosis.mp. [mp=title, abstract, original title, name of substance word, subject heading word, floating sub-heading word, keyword heading word, protocol supplementary concept word, rare disease supplementary concept word, unique identifier, synonyms] 7. MeSH HEADING: exp Arthralgia/ 8. (joint adj pain*).mp. [mp=title, abstract, original title, name of substance word, subject heading word, floating sub-heading word, keyword heading word, protocol supplementary concept word, rare disease supplementary concept word, unique identifier, synonyms] 9. arthrosis.mp. [mp=title, abstract, original title, name of substance word, subject heading word, floating sub-heading word, keyword heading word, protocol supplementary concept word, rare disease supplementary concept word, unique identifier, synonyms] 10. arthroses.mp. [mp=title, abstract, original title, name of substance word, subject heading word, floating sub-heading word, keyword heading word, protocol supplementary concept word, rare disease supplementary concept word, unique identifier, synonyms] 11. arthropath*.mp. [mp=title, abstract, original title, name of substance word, subject heading word, floating sub-heading word, keyword heading word, protocol supplementary concept word, rare disease supplementary concept word, unique identifier, synonyms] 12. **1 or 2 or 3 or 4 or 5 or 6 or 7 or 8 or 9 or 10 or 11** | Sets 1-11 are the MeSH terms and Text Word for the population (i.e people with osteoarthritis in any joint). They are combined using **OR** |
| **Exposure(s) (E)** | 1. exp Adaptation, Psychological/ 2. exp Adaptation, Physiological/ 3. exp adaptation, biological/ 4. Social Adjustment/ 5. exp Emotional Adjustment/ 6. adjustment.mp. [mp=title, abstract, original title, name of substance word, subject heading word, floating sub-heading word, keyword heading word, protocol supplementary concept word, rare disease supplementary concept word, unique identifier, synonyms] 7. exp Social Behavior/ 8. exp Psychology, Social/ 9. exp Psychology/ 10. exp Psychology, Industrial/ 11. exp Anxiety/ 12. DEPRESSION/ 13. Chronic Pain/ 14. Pain Management/ 15. exp Occupational Stress/ 16. exp Stress, Psychological/ 17. exp Fatigue/ 18. "Quality of Life"/ 19. coping.mp. [mp=title, abstract, original title, name of substance word, subject heading word, floating sub-heading word, keyword heading word, protocol supplementary concept word, rare disease supplementary concept word, unique identifier, synonyms] 20. coping behavio?r.mp. [mp=title, abstract, original title, name of substance word, subject heading word, floating sub-heading word, keyword heading word, protocol supplementary concept word, rare disease supplementary concept word, unique identifier, synonyms] 21. exp social environment/ 22. exp psychosocial support systems/ 23. Social Isolation/ 24. biopsychosocial.mp. [mp=title, abstract, original title, name of substance word, subject heading word, floating sub-heading word, keyword heading word, protocol supplementary concept word, rare disease supplementary concept word, unique identifier, synonyms] 25. bio-psychosocial.mp. [mp=title, abstract, original title, name of substance word, subject heading word, floating sub-heading word, keyword heading word, protocol supplementary concept word, rare disease supplementary concept word, unique identifier, synonyms] 26. biopsychosocial factors.mp. [mp=title, abstract, original title, name of substance word, subject heading word, floating sub-heading word, keyword heading word, protocol supplementary concept word, rare disease supplementary concept word, unique identifier, synonyms] 27. Economics, Behavioral/ 28. behavio?r*.mp. [mp=title, abstract, original title, name of substance word, subject heading word, floating sub-heading word, keyword heading word, protocol supplementary concept word, rare disease supplementary concept word, unique identifier, synonyms] 29. exp Socioeconomic Factors/ 30. socioeconomic.mp. [mp=title, abstract, original title, name of substance word, subject heading word, floating sub-heading word, keyword heading word, protocol supplementary concept word, rare disease supplementary concept word, unique identifier, synonyms] 31. socio-economic.mp. [mp=title, abstract, original title, name of substance word, subject heading word, floating sub-heading word, keyword heading word, protocol supplementary concept word, rare disease supplementary concept word, unique identifier, synonyms] 32. economic*.mp. [mp=title, abstract, original title, name of substance word, subject heading word, floating sub-heading word, keyword heading word, protocol supplementary concept word, rare disease supplementary concept word, unique identifier, synonyms] 33. MeSH HEADING: exp sociological factors/ 34. exp Ergonomics/ 35. Job Satisfaction/ 36. (job adj accommodation*).mp. [mp=title, abstract, original title, name of substance word, subject heading word, floating sub-heading word, keyword heading word, protocol supplementary concept word, rare disease supplementary concept word, unique identifier, synonyms] 37. (Workplace adj accommodation*).mp. [mp=title, abstract, original title, name of substance word, subject heading word, floating sub-heading word, keyword heading word, protocol supplementary concept word, rare disease supplementary concept word, unique identifier, synonyms] 38. (Work adj adaptation*).mp. [mp=title, abstract, original title, name of substance word, subject heading word, floating sub-heading word, keyword heading word, protocol supplementary concept word, rare disease supplementary concept word, unique identifier, synonyms] 39. (Work adj change*).mp. [mp=title, abstract, original title, name of substance word, subject heading word, floating sub-heading word, keyword heading word, protocol supplementary concept word, rare disease supplementary concept word, unique identifier, synonyms] 40. (Work adj transition*).mp. [mp=title, abstract, original title, name of substance word, subject heading word, floating sub-heading word, keyword heading word, protocol supplementary concept word, rare disease supplementary concept word, unique identifier, synonyms] 41. Occupational Health/ 42. exp Occupational Diseases/ 43. (Work adj environment).mp. [mp=title, abstract, original title, name of substance word, subject heading word, floating sub-heading word, keyword heading word, protocol supplementary concept word, rare disease supplementary concept word, unique identifier, synonyms] 44. (Work adj redesign).mp. [mp=title, abstract, original title, name of substance word, subject heading word, floating sub-heading word, keyword heading word, protocol supplementary concept word, rare disease supplementary concept word, unique identifier, synonyms] 45. Rehabilitation, Vocational/ 46. Workload/ 47. (work adj3 attitude*).mp. [mp=title, abstract, original title, name of substance word, subject heading word, floating sub-heading word, keyword heading word, protocol supplementary concept word, rare disease supplementary concept word, unique identifier, synonyms] 48. environmental adjustment.mp. [mp=title, abstract, original title, name of substance word, subject heading word, floating sub-heading word, keyword heading word, protocol supplementary concept word, rare disease supplementary concept word, unique identifier, synonyms] 49. Employment, Supported/ 50. burden.mp. [mp=title, abstract, original title, name of substance word, subject heading word, floating sub-heading word, keyword heading word, protocol supplementary concept word, rare disease supplementary concept word, unique identifier, synonyms] 51. impact.mp. [mp=title, abstract, original title, name of substance word, subject heading word, floating sub-heading word, keyword heading word, protocol supplementary concept word, rare disease supplementary concept word, unique identifier, synonyms] 52. **13 or 14 or 15 or 16 or 17 or 18 or 19 or 20 or 21 or 22 or 23 or 24 or 25 or 26 or 27 or 28 or 29 or 30 or 31 or 32 or 33 or 34 or 35 or 36 or 37 or 38 or 39 or 40 or 41 or 42 or 43 or 44 or 45 or 46 or 47 or 48 or 49 or 50 or 51 or 52 or 53 or 54 or 55 or 56 or 57 or 58 or 59 or 60 or 61 or 62 or 63** | Sets 13-63 are the MeSH terms and Text Word for the exposures (i.e any biopsychosocial factors that could impact work participation). They are combined using **OR** |
| **Outcome (O)** | 1. Exp Work/ 2. Workplace/ 3. exp Employment/ 4. vocation.mp. [mp=title, abstract, original title, name of substance word, subject heading word, floating sub-heading word, keyword heading word, protocol supplementary concept word, rare disease supplementary concept word, unique identifier, synonyms] 5. (paid adj work).mp. [mp=title, abstract, original title, name of substance word, subject heading word, floating sub-heading word, keyword heading word, protocol supplementary concept word, rare disease supplementary concept word, unique identifier, synonyms] 6. Return to Work/ 7. exp Occupations/ 8. Occupation*.mp. [mp=title, abstract, original title, name of substance word, subject heading word, floating sub-heading word, keyword heading word, protocol supplementary concept word, rare disease supplementary concept word, unique identifier, synonyms] 9. exp Disability Evaluation/ 10. Sick Leave/ 11. Absenteeism/ 12. Presenteeism/ 13. exp EFFICIENCY/ 14. exp "Task Performance and Analysis"/ 15. Work Performance/ 16. (job adj performance).mp. [mp=title, abstract, original title, name of substance word, subject heading word, floating sub-heading word, keyword heading word, protocol supplementary concept word, rare disease supplementary concept word, unique identifier, synonyms] 17. (work adj participation).mp. [mp=title, abstract, original title, name of substance word, subject heading word, floating sub-heading word, keyword heading word, protocol supplementary concept word, rare disease supplementary concept word, unique identifier, synonyms] 18. (work adj productivity).mp. [mp=title, abstract, original title, name of substance word, subject heading word, floating sub-heading word, keyword heading word, protocol supplementary concept word, rare disease supplementary concept word, unique identifier, synonyms] 19. (work adj impairment).mp. [mp=title, abstract, original title, name of substance word, subject heading word, floating sub-heading word, keyword heading word, protocol supplementary concept word, rare disease supplementary concept word, unique identifier, synonyms] 20. (work adj limitation*).mp. [mp=title, abstract, original title, name of substance word, subject heading word, floating sub-heading word, keyword heading word, protocol supplementary concept word, rare disease supplementary concept word, unique identifier, synonyms] 21. (job adj limitation*).mp. [mp=title, abstract, original title, name of substance word, subject heading word, floating sub-heading word, keyword heading word, protocol supplementary concept word, rare disease supplementary concept word, unique identifier, synonyms] 22. (job adj disruption*).mp. [mp=title, abstract, original title, name of substance word, subject heading word, floating sub-heading word, keyword heading word, protocol supplementary concept word, rare disease supplementary concept word, unique identifier, synonyms] 23. (work adj disruption*).mp. [mp=title, abstract, original title, name of substance word, subject heading word, floating sub-heading word, keyword heading word, protocol supplementary concept word, rare disease supplementary concept word, unique identifier, synonyms] 24. Retirement/ 25. (early adj retirement).mp. [mp=title, abstract, original title, name of substance word, subject heading word, floating sub-heading word, keyword heading word, protocol supplementary concept word, rare disease supplementary concept word, unique identifier, synonyms] 26. **65 or 66 or 67 or 68 or 69 or 70 or 71 or 72 or 73 or 74 or 75 or 76 or 77 or 78 or 79 or 80 or 81 or 82 or 83 or 84 or 85 or 86 or 87 or 88 or 89** | Sets 65-89 are the MeSH terms and Text Word for the outcomes related to work participation. They are combined using **OR** |
| **Study design (SD)** | 1. exp epidemiologic studies/ or exp case-control studies/ or exp cohort studies/ or cross-sectional studies/ 2. "surveys and questionnaires"/ or exp health care surveys/ or exp health surveys/ 3. **91 or 92** | Sets 91-92 are the MeSH terms and Text Word for the study design. They are combined using **OR** |
| **Combination of P & E & O & SD** | 1. **12 and 64 and 90 and 93** | Final combination:  12 and 64 and 90 and 93 |
