# Supplementary material for: Biopsychosocial, work-related, and environmental factors affecting work participation in people with Osteoarthritis: a systematic review
Source: BMC Musculoskelet Disord. 2023 Jun 13;24:485. doi: 10.1186/s12891-023-06612-6 (PMC10262400; doi:10.1186/s12891-023-06612-6)
Supplement: Supplementary file 2 — Additional file 2. Joanna Briggs Critical Appraisal Tool items: Cohort and Cross-sectional studies. [file 12891_2023_6612_MOESM2_ESM.docx]

Article title: Biopsychosocial, work-related, and environmental factors affecting work participation in people with Osteoarthritis: A systematic review.

Journal name: BMC Musculoskeletal Disorders

Authors: Angela Ching^1^, Yeliz Prior^1^, Jennifer Parker^1^, Alison Hammond^1^

Affiliation: ^1^Centre for Health Sciences Research, University of Salford, Salford, Greater Manchester, United Kingdom.

Corresponding Author: Professor Yeliz Prior (email: [y.prior@salford.ac.uk](mailto:y.prior@salford.ac.uk))

**Additional File 2: Joanna Briggs Critical Appraisal Tool items: Cohort and Cross-sectional studies.**

Cohort studies:

1. Were the two groups similar and recruited from the same population?
2. Were the exposures measured similarly to assign people to both exposed and unexposed groups?
3. Was the exposure measured in a valid and reliable way?
4. Were confounding factors identified?
5. Were strategies to deal with confounding factors stated?
6. Were the groups/participants free of the outcome at the start of the study (or at the moment of exposure)?
7. Were the outcomes measured in a valid and reliable way?
8. Was the follow up time reported and sufficient to be long enough for outcomes to occur?
9. Was follow up complete, and if not, were the reasons to loss to follow up described and explored?
10. Were strategies to address incomplete follow up utilized?
11. Was appropriate statistical analysis used?

Cross-sectional studies:

1. Were the criteria for inclusion in the sample clearly defined?
2. Were the study subjects and the setting described in detail?
3. Was the exposure measured in a valid and reliable way?
4. Were objective, standard criteria used for measurement of the condition?
5. Were confounding factors identified?
6. Were strategies to deal with confounding factors stated?
7. Were the outcomes measured in a valid and reliable way?
8. Was appropriate statistical analysis used?
