# Supplementary material for: Biopsychosocial, work-related, and environmental factors affecting work participation in people with Osteoarthritis: a systematic review
Source: BMC Musculoskelet Disord. 2023 Jun 13;24:485. doi: 10.1186/s12891-023-06612-6 (PMC10262400; doi:10.1186/s12891-023-06612-6)
Supplement: Supplementary file 3 — Additional file 3. Excluded studies after the quality assessment process. [file 12891_2023_6612_MOESM3_ESM.docx]

Article title: Biopsychosocial, work-related, and environmental factors affecting work participation in people with Osteoarthritis: A systematic review.

Journal name: BMC Musculoskeletal Disorders

Authors: Angela Ching^1^, Yeliz Prior^1^, Jennifer Parker^1^, Alison Hammond^1^

Affiliation: ^1^Centre for Health Sciences Research, University of Salford, Salford, Greater Manchester, United Kingdom.

Corresponding Author: Professor Yeliz Prior (email: [y.prior@salford.ac.uk](mailto:y.prior@salford.ac.uk))

**Additional File 3: Excluded studies after the quality assessment process**

All were cohort studies.

| **Author, year, country** | **Study aims** | **Study design and data** | **Study population** | **Relevant outcome measures** | **Reason(s) for exclusion at the quality assessment process** |
| --- | --- | --- | --- | --- | --- |
| Jorn *et al.,* 1999, Sweden [28] | To investigate working status 2 years pre- and 2 years post- primary knee prosthetic operation for arthrosis to identify factors predicting patient satisfaction, function and  working capacity. | Cohort study, data from the Social Insurance Office and questionnaires | Adults with knee OA; ≤60 years when operated on in Sweden in 1993 (identified through the Swedish National Knee Register). | Return to work: no clear definition; sick leave: measured pre-and post-operative (<180 days or ≥180 days in 2 years pre- and 2 years post-operative period); working status: working, 50% disability pension or 100% disability pension (pre- and post-operative periods) | Quality: 55% (moderate). Unclear if exposures measured similar in both exposed and unexposed groups; confounders not reported and strategies to deal with these not stated, meaning results may be biased by other differences between groups. Reasons for missing data/missing follow-ups not explored or reported meaning no demographic data in ‘missing/non-responders’ group. |
| Wolf *et al.,* 2018, Sweden [29] | To evaluate amount of sick leave associated with surgery for thumb carpometacarpal (CMC1) OA, using Swedish Registry surgical records, stratified by type of procedure, cross-linked with individual-level sick leave data. | Cohort study - secondary data analysis using Registry data from the Skåne region, Sweden, cross-linked with employment data showing person-specific sick leave | Skåne region, Sweden | Sick leave: obtained from the Swedish Social Insurance Agency | Quality: 73% (moderate). Sick leave was measured irrespective of cause. Thus, we cannot that all days of sick leave are attributable to the joint surgery. Only those with sick leave for >14 days are registered. Those with sick leave <14 days are not registered.  Unclear if participants free of the outcome prior to study start or at point of exposure. Some participants were on sick leave prior to surgery, so were not free of the outcome at the start of the study. |
| Hoorntje *et al.,* 2019, The Netherlands [27] | To investigate extent and timing of return to work (RTW) in a high tibial osteotomy (HTO) cohort and to identify prognostic factors for RTW after HTO. | Cohort study | Consecutive patients who underwent HTO between 2012 -2015 | Percentage of patients who RTW postoperatively and timing of RTW. Patients were asked if worked before onset of restricting knee symptoms and in the 3 months prior to surgery. | Quality: 45.5% (low). A convenience sample was used to obtain a large cohort, but unclear how similar patients were in terms of other health (e.g., comorbidities) and social demographics, other than having undergone HTO, aged 18-60 years, with BMI <30 kg/m2, speaking Dutch and being able to complete a questionnaire.  Diagram of causal assumptions for selecting covariates unclear, and assumptions behind including variables into analysis model had insufficient justification, e.g., ‘being self-employed is hypothesized to increase patients’ motivation, positively influencing RTW’ was not substantiated with evidence. This introduced uncertainty about reliability of the statistical analysis is. Authors identified the use of prognostic factors (e.g., OA or non-OA groups) might be problematic. |

^Key: OA = osteoarthritis; CMC1 = thumb carpometacarpal; RTW = return to work; HTO = high tibial osteotomy.^
