# Supplementary material for: Biopsychosocial, work-related, and environmental factors affecting work participation in people with Osteoarthritis: a systematic review
Source: BMC Musculoskelet Disord. 2023 Jun 13;24:485. doi: 10.1186/s12891-023-06612-6 (PMC10262400; doi:10.1186/s12891-023-06612-6)
Supplement: Supplementary file 4 — Additional file 4. Outcomes: Work participation. [file 12891_2023_6612_MOESM4_ESM.docx]

Article title: Biopsychosocial, work-related, and environmental factors affecting work participation in people with Osteoarthritis: A systematic review.

Journal name: BMC Musculoskeletal Disorders

Authors: Angela Ching^1^, Yeliz Prior^1^, Jennifer Parker^1^, Alison Hammond^1^

Affiliation: ^1^Centre for Health Sciences Research, University of Salford, Salford, Greater Manchester, United Kingdom.

Corresponding Author: Professor Yeliz Prior (email: [y.prior@salford.ac.uk](mailto:y.prior@salford.ac.uk))

**Additional File 4 Outcomes: Work participation**

| **Author, year, country** | **Absenteeism** | **Presenteeism** | **Work transitions/ impairment / difficulty / limitations** | **Biopsychosocial factors and associations with outcomes** |
| --- | --- | --- | --- | --- |
| Agaliotis *et al.*, 2013, Australia [24]  (see Table 5 for data on work accommodations) | 14%: ≥1 days off work due to knee problems. | 79%: reduced work productivity at; 42% of whom <90% presenteeism due to knee problems at 12m. follow-up. |  | Multivariate analysis. Risk factors:  ≥ 1 day absenteeism due to knee problems at 12-m. follow-up: SF-12 MCS <40 at baseline vs ≥50 (OR: 2.49, 95% CI: 1.03–5.98).  Reduced work productivity: SF-12 PCS <50 at baseline (OR: 1.99, 95% CI: 1.05–3.76), working in semi-manual (OR: 2.23, 95% CI: 1.09–4.59) or manual labour (OR: 6.40, 95% CI: 1.44–28.35) and knee pain score 4 to 6 (OR: 2.29, 95% CI: 1.17–4.46). |
| Agaliotis *et al.*, 2017, Australia [36] | 5%: ≥1 whole days off work due to knee problems. | 49%: some reduced worker productivity at work due to knee problems. | 24% ≥1 work transitions due to knee problems. Most common = work interrupted at least 20mins (15%), unable to take on extra projects/ responsibilities (11%), and lost time at work (leaving work early, arriving late, or taking an extended lunch hour) (9%). | Multivariate analysis. Factors associated with presenteeism: moderate-to-severe knee pain (≥3/10) in past week (OR: 2.77, 95% CI: 1.30–5.87), problems with ≥1 other joints (OR: 2.32, 95% CI: 1.04–5.17), or reporting job insecurity (OR: 0.40, 95% CI: 0.19–0.86).  Factors associated with work transitions: moderate-to-severe knee pain (≥3/10) in past week (OR: 4.09, 95% CI: 1.53–10.95), comorbidity score ≥4 (OR: 4.44, 95% CI: 1.02–19.32), or low co-worker support (OR: 2.79, 95% CI: 1.04–7.46).  Univariate/multivariate modelling were not performed for absenteeism. |
| Bieleman *et al.*, 2010, The Netherlands [13]  (see Table 5 for data on work accommodations) | CHECK Cohort: absenteeism = 7.7%, 10 because of hip/knee symptoms (point prevalence of 2.0% of the workers). Past 12 m.: 61 participants reported absenteeism due to hip/knee symptoms (year prevalence 12.4%). | --- | --- | -- |
| Conaghan *et al.*, 2021, Europe, [37] |  | Mild pain + prescription medication-treated group significantly higher presenteeism (47.2%) vs moderate/severe pain and no prescription medication group (43.9%) (p<0.001) | Moderate/severe pain + prescription medication-treated group 2–6x higher work impairment vs mild pain and no prescription medication group (p<0.001), and respondents in the other pain and treatment groups reported about 1.5–2x greater work productivity and activity impairment compared with the mild pain untreated with prescription medication group (p<0.001) | Healthcare resource use (past 6m) significantly higher in prescription medication groups vs no prescription medication groups (p<0.05). |
| daCosta DiBonaventura *et al.*, 2011, USA [38] | Workers with OA pain = 8% time lost through absenteeism (i.e., 2.7 (SD 7.1) hours in past week) vs. participants without OA pain = 4% (1.4 (SD 5.6) hours). | Workers with OA pain lost 31% of productive time at work (9.7 (SD 9.7) h.) vs. participants without OA pain lost 16% (5.2 (SD 8.6) h.). | OA pain cohort greater work impairment than those without OA pain (34.4% vs. 17.8%), primarily due to presenteeism. | Health status significantly lower in workers with OA pain: PCS =7.6 points and MCS = 1.2 points lower than in no-OA pain cohort. |
| daCosta DiBonaventura *et al.*, 2012, USA [39] | Hours lost: workers with mild OA = (1.6 (SD 5.7) h., moderate OA = 2.5 (SD 7.1) h., severe OA = 4.8 (SD 9.7) h. vs workers with no OA = 1.3 (SD 5.6) h. | Hours lost due to presenteeism: mild OA = 5.86 (SD 8.1) h; moderate OA = 9.37 (SD 9.6) h., severe OA = 12.5 (SD 12.0) h. vs workers without OA = 5.05 (SD 8.5) h. | Overall work impairment: OA pain group 34.4% vs no OA pain group 17.8%.  Workers with moderate and severe OA: significantly higher percentages of overall work impairment, and activity impairment vs workers without OA. No differences between for mild OA group. | Workers with mild, moderate, and severe OA: significantly worse SF-12v2 PCS scores as OA severity level increased; MCS scores significantly increased in mild OA group vs. non-OA workers. |
| Hermans *et al.*, 2012, The Netherlands [40] | Knee-related absence from work = 6.6 h./ month, costing €197/patient/month. | Knee-related lost productivity at work (14.9 hours/month), costing €448/ patient/ month. Accounted for 62% of total productivity-loss costs. | --- | Multivariate linear regression Factors associated with: Presenteeism: pain during activity (b: -0.28, 95% CI: -0.47–0.09) and performing physically intensive work (b: -1.73, 95% CI: -2.62–0.84).  Absenteeism: performing physically intensive work (OR: 4.20, 95% CI: 1.48–11.93). |
| Hubertsson *et al.*, 2013, Sweden [14]  (see Table 4 for data on disability pension) | Risk of absenteeism increased in all age categories for both genders. Overall age-standardised and sex-specific RR vs general population = women: 1.82 (95% CI: 1.73–1.91); men: 2.03 (95% CI: 1.92–2.14).  Six-year period, no. days absenteeism and/or disability pension (irrespective of cause): People diagnosed with knee OA = 87 (SD 140) days.  General population (age- and sex- standardised) = 57 (SD 78).  Absenteeism: no. days/year:  Women with knee OA: 18 (SD 57) days.  Men with knee OA: 15 (SD 52).  1.4% (1.2% women; 1.6% men) of all net days with absenteeism due to knee OA. | --- | --- | --- |
| Hubertsson *et al.*, 2017, Sweden [30]  (see Table 4 for data on disability pension) | Risk of absenteeism increased (adjusted for age and education):  **Knee OA:**  **Women in** health care sector (OR: 3.27, 95% CI: 2.58–4.14); childcare (OR: 2.97, 95% CI: 2.25–3.90); and cleaning (OR: 3.07, 95% CI: 2.24–4.21) vs business/administration sector. Occupations with higher educational requirements in health care (specialist nurses) (OR: 2.80, 95% CI: 1.90–4.20) and childcare (pre-primary teaching professionals) (OR: 2.70, 95% CI: 1.80–4.10) at higher risk.  **Men in** construction (OR: 2.97, 95% CI: 2.25–3.92); farming (OR: 1.67, 95% CI: 1.12–2.49); metal work (OR: 2.57, 95% CI: 1.69–3.90); and transportation (OR: 1.81, 95% CI: 1.29–2.53).  **Hip OA:**  **Women:** occupation not associated with increased risk of absenteeism.  **Men:** occupation was associated with slight increase in absenteeism for men in construction (OR: 1.46, 95% CI: 1.06–2.00) and farming (OR: 1.56, 95% CI: 1.03–2.38). | --- | --- | --- |
| Jackson *et al.*, 2020, Europe (EU) and USA [41] | Participants with:  Moderate/ severe pain (WOMAC 4-10 score) without opioid use had greater absenteeism than those with no/mild pain (WOMAC 0-3) (WPAI: 20.5% (SD 36.7) vs 5.5% (SD 17.4)).  Moderate/ severe pain with opioid use had greater absenteeism than those with no/mild pain (WPAI: 22.4% (SD 37.0) vs 8.2% (SD 22.5)).  Moderate/ severe pain using opioids: absenteeism more evident in EU than US cohort (WPAI: 33.3% (SD 41.6) vs 6.3% (SD 21.1)). | Participants with:  Moderate/ severe pain without opioid use: greater work impairment due to presenteeism than no/mild pain (WPAI: 47.7% (SD 22.4) vs 21.6% (SD 17.9)).  Moderate/ severe pain with opioid use: greater work impairment due to presenteeism than those with no/mild pain (WPAI: 51.5 (SD 21.2) vs 27.8 (SD 19.8), respectively).  Moderate/severe pain with/ without opioid use: significantly greater work productivity impairment, compared to no/mild pain without opioid use (p<0.05). | Participants with moderate/ severe pain: greater overall work impairment than those with no/mild pain.  Percentage overall work impairment substantially greater in EU than US cohort. | --- |
| Kontio et al., 2020, Finland [34] | Annual proportion of absenteeism due to OA (mean time for all persons year) during first year of follow-up similar for knee OA; hip OA; polyarthritis or CMC joint OA; and other OA group (i.e., 3.4%, 3.6%, 3.9%, 2.9%). | -- | On average, 5.9 transitions (95% CI: 5.8–6.0) between different work participation statuses per person. | -- |
| Nakata *et al.*, 2018, Japan [43] | Absenteeism in past week = 11.1% of participants  More patients with than without presenteeism reported absenteeism (15.1% vs. 1.5%, p=0.001).  Absenteeism and overall work impairment greater in those with than without presenteeism (absenteeism: 2.9% ± 10.8% vs. 0.0% ± 0.4%,  p=0.034; overall work impairment: 39.5% ± 25.1% vs. 0.0% ± 0.4%, p<0.001). | Presenteeism in past week =71.2% of participants. | --- | Absenteeism associated with  Younger age (any = 46.1 ± 15.3 years vs none = 55.4 ± 11.3 years: p<0.001).  Higher comorbidity (CCI) scores (any = 3.1 ± 8.0 vs none = 0.7 ± 3.3; p=0.006).  Presenteeism associated with:  Younger age (any = 52.9 ± 12.4 years vs none = 57.3 ± 11.2 years, p=0.012).  Higher presenteeism moderately associated with lower MCS (r_s_ = -0.44), PCS (r_s_ = -0.46), and SF-6D (r_s_ = -0.51) scores (all p<0.001).  Higher presenteeism moderately associated with greater depression severity (r_s_ = 0.42, p<0.001).  Greater depression symptom severity higher (PHQ-9 mean (SD): 5.8 ± 6.0) vs those without presenteeism (2.9 ± 4.3, p<0.001).  Lower proportion married/ living with a partner (any = 62.7% vs. none = 76.1%, p=0.049); and had greater educational attainment vs no presenteeism.  Prescription medications use higher in those with presenteeism (37.3%) vs without (20.9%, p=0.015).    Greater depression symptom severity higher (PHQ-9 mean (SD): 5.8 ± 6.0) vs those without presenteeism (2.9 ± 4.3, p<0.001). |
| Summanen *et al.*, 2021, Finland [35] | OA patients: 2.8x absenteeism vs controls (22.8 vs 8.1 days per patient year (PPY)) and 2.2x more periods of absenteeism (2.2 vs 1.0 days PPY). For the occupational healthcare (OCH) sub-cohort OA patients, 28% of all absenteeism days were recorded with an OA diagnosis. Overall, 78.9% of the OCH OA patients had at least one day of absenteeism per year vs 60% of controls. | --- | --- | Hip/knee OA patients with normal BMI (≤ 25 kg/m2): 16.3 days absenteeism PPY.  Patients with a BMI > 30 kg/m2: 28.2 days absenteeism PPY.  Same trend for controls, but overall values lower (7.3 vs 9.4 days absenteeism PPY for BMI ≤ 25 kg/m2 and BMI > 30 kg/m2, respectively).  Comorbidities (e.g., type 2 diabetes) increased absenteeism from 21.7 to 31.2 days PPY for hip/ knee OA patients; and from 7.9 to 12.8 days PPY for controls.  Both hip/knee OA patients and controls with COPD had more absenteeism days vs those without COPD (39.0 vs 22.3 days PPY, and 20.3 vs 8.1 days PPY for controls with and without COPD, respectively). |
| Wilkie *et al.*, 2014, United Kingdom [33] | --- | --- | 17.2% indicated joint pain will not limit ability to work until 69y; 26.6% indicated expected work limitation (EWL); 56.2% did not know if joint pain will limit work before 69y. | Multivariate analysis. Factors associated with EWL:  physical function (OR: 0.95; 95% CI: 0.92 to 0.97); age (OR: 0.74; 95% CI: 0.60 to 0.91).  Better physical function score was protective against EWL onset prior to 69 y.  6.7% of participants reported low co-worker support, all of whom predicted developing EWL before age 69 years. |
| Wilkie *et al.*, 2015, United Kingdom [32] | --- | 16.7% reported onset of work productivity loss | --- | High pain intensity at baseline significantly associated with onset of work productivity loss three years later, after adjusting for age, gender, educational attainment, occupational class, and comorbidity (OR: 2.50, 95% CI: 1.30–4.80).  Physical limitation mediated association between pain intensity and onset of work productivity loss. Depression, poor sleep quality and poor coping mechanisms did not. |

^Key: CMC = carpometacarpal; COPD = chronic obstructive pulmonary disease; OA = osteoarthritis;^ **^Data source^**^: CHECK = the Cohort Hip and Cohort Knee, OAI = Osteoarthritis Initiative; OCH = occupational healthcare sub-cohort.^ **^Measures^**^: CCI = Charlson Comorbidity Index; EWL = expected work limitation; PHQ-9 = Patient Health Questionnaire-9;^ ^SF36v2 = Medical Outcomes Survey Short Form 36 item v2; SF-6D = Medical Outcomes Survey Short Form Six Items; MCS = Mental Component Summary score SF-36v2; PCS = Physical Component Summary score SF-36v2; WOMAC = Western Ontario and McMaster Universities Osteoarthritis Index;^ ^WPAI = Work Productivity and Activity Impairment.^ **^Other^**^: 95% CI = 95% confidence interval; BMI = body mass index; h = hour(s); no. = number; OR = Odds ratio; PPY = per patient year; SD = standard deviation; vs = versus; y = years.^
