# Supplementary material for: Biopsychosocial, work-related, and environmental factors affecting work participation in people with Osteoarthritis: a systematic review
Source: BMC Musculoskelet Disord. 2023 Jun 13;24:485. doi: 10.1186/s12891-023-06612-6 (PMC10262400; doi:10.1186/s12891-023-06612-6)
Supplement: Supplementary file 5 — Additional file 5. Outcomes: leaving work before statutory retirement age. [file 12891_2023_6612_MOESM5_ESM.docx]

Article title: Biopsychosocial, work-related, and environmental factors affecting work participation in people with Osteoarthritis: A systematic review.

Journal name: BMC Musculoskeletal Disorders

Authors: Angela Ching^1^, Yeliz Prior^1^, Jennifer Parker^1^, Alison Hammond^1^

Affiliation: ^1^Centre for Health Sciences Research, University of Salford, Salford, Greater Manchester, United Kingdom.

Corresponding Author: Professor Yeliz Prior (email: [y.prior@salford.ac.uk](mailto:y.prior@salford.ac.uk))

**Additional File 5 Outcomes: leaving work before statutory retirement age**

| **Author, year, country** | **Leaving work before statutory retirement age** | **Biopsychosocial factors and associations with outcomes** |
| --- | --- | --- |
|  | 1. **Premature work loss / early exit from work / early retirement** |  |
| Wilkie *et al.*, 2014, United Kingdom [17] | 23.6% with premature work loss (PWL). Primary care OA consulters off work due to sickness vs those without OA (33.8% vs 19.1%). | Male (OR: 1.97, 95% CI: 1.28–3.04), pain interference (OR: 1.51, 95% CI: 1.00–2.27) and low co-worker support (OR: 3.11, 95% CI: 1.78–5.42) independently associated with PWL in OA consulters. |
| Laires *et al.*, 2018, Portugal [42] | OA associated with: PWL (OR: 1.85, 95% CI: 1.27–2.69); but not official early retirement (OR: 1.43, 95% CI: 0.96–2.12).  Knee OA strongly associated with PWL (OR: 2.25, 95% CI: 1.42–3.59). No significant association hand or hip OA.  Unemployment associated with PWL (OR: 1.97, 95% CI: 1.27–3.06), especially knee OA (OR: 2.68, 95% CI: 1.58–4.53), and younger age (50–57) (OR: 3.47, 95% CI: 1.88–6.41). | Strong association pain interference and PWL, especially knee OA (OR: 1.52, 95% CI: 1.16–1.99).  Knee OA: highest levels of disability (HAQ scores ≥ 2) at greatest risk PWL. |
| Kontio *et al.*, 2020, Finland [34] | On average 2.1 (95% CI: 2.0–2.2) potential working life–years lost. Those with polyarthritis/CMC joint OA lost significantly more working life–years (2.5, 95% CI: 2.3–2.5) than those with knee or hip OA (2.0, 95% CI: 1.9–2.1 and 2.0, 95% CI: 1.8–2.1, respectively). | Univariate model PWL predictors: male, older age, low education, long initial sickness absence, not returned to work sustainably after the initial sickness absence and receiving vocational rehabilitation. All physical work-related factors increased risk of PWL, but not significant in multivariate model. |
|  | 1. **Disability pension / disability retirement** |  |
| Kontio et al., 2020, Finland [34] | Annual proportion time spent in permanent disability retirement (mean time all persons/year) highest in polyarthritis/CMC joint OA group vs knee, hip, and other OA 17.7% vs 12.1%, 11.1% 12.0%, respectively. |  |
| Hubertsson *et al.*, 2013, Sweden [14]  (see Table 3 for data on absenteeism) | **Knee OA:** 21% women; 17% men received sickness benefit during previous year.  More women than men received disability pension payment (32% vs 16%).  Disability pension payment increased with age.  Women = 94 (SD 149) days disability pension/person/year.  Men = 47 (SD 115) days disability pension/person/year. | **---** |
| Hubertsson *et al.*, 2017, Sweden [30]  (see Table 3 for data on absenteeism) | Risk of disability pension (adjusted for age and education):  Knee OA increased:   - in all job sectors vs business and administration. - for women in health care (OR: 10.25, 95% CI: 4.78–21.97), childcare (OR: 9.83, 95% CI: 4.36–22.17), and cleaning (OR: 16.70, 95% CI: 7.46–37.37). - men in construction (OR: 3.45, 95% CI: 1.96–6.09), metal work (OR: 4.35, 95% CI: 2.09–9.02), and transportation (OR: 2.09, 95% CI: 1.06–4.10).   Hip OA increased:   - in all studied job sectors vs business and administration. - for women in health care (OR: 6.91, 95% CI: 2.49–19.13), childcare (OR: 5.89, 95% CI: 1.92–18.07), and cleaning (OR: 5.44, 95% CI: 1.50–19.72). - men in farming (OR: 3.15, 95% CI: 1.16–8.55). | --- |
| Kontio *et al.*, 2018, Finland [31] | No. full disability retired due to knee OA = 6117.  Overall age-adjusted incidence = 60 (men); 72 (women)/ 100,000 person-years. | Physical work-related factors and disability retirement adjusted for age: all physical load factors (heavy physical work; kneeling/squatting ≥1 hr/day; heavy lifting ≥20kg, ≥10 times/day; sitting ≥5 hrs/day; standing or moving ≥5 hrs/day) statistically significantly associated with disability retirement due to knee OA in men (range: sitting (HR: 0.28, 95% CI: 0.25–0.32) to standing or moving (HR: 2.52, 95% CI: 2.32–2.73) and women (range: sitting HR: 0.23, 95% CI: 0.20–0.26) to heavy physical work (HR 2.75, 95% CI 2.57–2.95)). All physical load factors increased risk disability retirement apart from sitting which reduced risk.  High risk disability retirement. Men: service workers, electricians, plumbers, construction workers, unskilled transport, construction, and manufacturing workers. Women: assistant nurses, building caretakers, cleaners, kitchen workers. Risk in manual workers strongly attributed to physically heavy work. |

^Key: CMC = carpometacarpal; OA = osteoarthritis;^ **^Measures^**^: HAQ = Health Assessment Questionnaire; PWL = premature work loss (i.e., leaving work or losing job prior to State Pension age);^ **^Other^**^: 95% CI = 95% confidence interval; HR = Hazard ratio; no. = number; OR = Odds ratio; SD = standard deviation; vs = versus.^
