# Supplementary material for: Biopsychosocial, work-related, and environmental factors affecting work participation in people with Osteoarthritis: a systematic review
Source: BMC Musculoskelet Disord. 2023 Jun 13;24:485. doi: 10.1186/s12891-023-06612-6 (PMC10262400; doi:10.1186/s12891-023-06612-6)
Supplement: Supplementary file 6 — Additional file 6. Outcomes: workplace accommodations and adaptations. [file 12891_2023_6612_MOESM6_ESM.docx]

Article title: Biopsychosocial, work-related, and environmental factors affecting work participation in people with Osteoarthritis: A systematic review.

Journal name: BMC Musculoskeletal Disorders

Authors: Angela Ching^1^, Yeliz Prior^1^, Jennifer Parker^1^, Alison Hammond^1^

Affiliation: ^1^Centre for Health Sciences Research, University of Salford, Salford, Greater Manchester, United Kingdom.

Corresponding Author: Professor Yeliz Prior (email: [y.prior@salford.ac.uk](mailto:y.prior@salford.ac.uk))

**Additional File 6 Outcomes: workplace accommodations and adaptations**

| **Author, year, country** | **Workplace accommodations and adaptations** | **Biopsychosocial factors and associations with outcomes** |
| --- | --- | --- |
| Bieleman *et al.*, 2010, The Netherlands [13]  (see Table 3 for data on absenteeism) | Due to hip/knee symptoms:  Fewer hours (38%); work technique (27%); work aids/place (25%); other/fewer tasks (10%)  Desired adaptations:  Fewer hours (35%); work aids/place (27%); other/fewer tasks (24%); work technique (14%) | --- |
| Gignac *et al.*, 2018, Canada [25] | More women needed 5+ accommodations vs. men (36.2% vs 19.8%, respectively).  No differences men and women in use of accommodation (p=0.33). Women more likely to report help with job tasks than men.  More often reported using an accommodation than said they need it (i.e., needs exceeded). | Need for 2–4 accommodations (vs 0–1) predicted by greater: workplace activity limitations (OR: 1.29) and health variability (OR: 1.74). Women more likely to report needing ≥5 accommodations than men.  ≥5 Accommodation use predicted by: workplace activity limitations (OR: 1.16), physical work demands (OR: 0.70), and health variability (OR: 1.69) vs 0–1 accommodation use.  Unmet accommodation needs: more likely to work in sales/retail jobs (OR: 3.73), greater work stress (OR: 1.54), and less job control (OR: 0.93) vs those whose accommodation needs met.  Accommodation needs exceeded: greater job control (OR: 1.13) vs those with unmet accommodation needs. |
| Agaliotis et al., 2013, Australia [24]  (see Table 3 for data on absenteeism) | At 12-m: 99 participants made at least one change to work: changing occupation (n=43); increasing (n=21) or decreasing (n=20) work hours; 10 retired (7 at ≥ 65 years) and 5 lost jobs (unrelated to knee problems). |  |

^Key: m. = month(s); OR = Odds ratio; vs = versus.^
